# Severe problem of macrolides resistance to common pathogens in China

**DOI:** 10.3389/fcimb.2023.1181633

**Published:** 2023-08-10

**Authors:** Jialin Li, Lesen Liu, Hua Zhang, Jing Guo, Xiaoling Wei, Min Xue, Xiang Ma

**Affiliations:** ^1^ Department of Respiratory Disease, Children's Hospital Affiliated to Shandong University, Jinan, Shandong, China; ^2^ Jinan Key Laboratory of Pediatric Respiratory Diseases, Jinan Children’s Hospital, Jinan, China; ^3^ Surgical Department, Huaiyin People’s Hospital, Jinan, China

**Keywords:** resistance, macrolides, bacteria, child, China

## Abstract

With the widespread use of macrolide antibiotics in China, common pathogens causing children’s infections, such as *Streptococcus pneumoniae*, *Streptococcus* (including *Group A streptococcus*, *Group B streptococcus*), *Staphylococcus aureus*, *Bordetella pertussis*, and *Mycoplasma pneumoniae*, have shown varying degrees of drug resistance. In order to provide such problem and related evidence for rational use of antibiotics in clinic, we reviewed the drug resistance of common bacteria to macrolides in children recent 20 years.

## Introduction

Macrolides (MLs) are a diverse class of hydrophobic compounds characterized by a macrocyclic lactone ring that typically contains at least 12 elements and distinguished by variable side chains/groups. The 23S rRNA in 50S subunit in bacterial ribosome contains a peptidyl transferase (PTC) that catalyzes peptide bond formation to link amino acids into proteins. MLs interact with the nucleic acid in the domain V of the catalytic center of the enzyme, bind to the part between PTC and nascent peptide exit tunnel and then block the channel to inhibit the synthesis of bacterial proteins, which finally playing an antibacterial effect (Vázquez-Laslop and Mankin, 2018). In addition to antibiotic properties, MLs also have been shown to display antiviral, antiparasitic, antifungal, and immunosuppressive actions. So, they were widely used in clinical.

However, with the widespread use of these drugs, many bacteria are showing a tendency to become more resistant to the drugs. The mechanisms of ML resistance mainly include the following: 1.erm-mediated rRNA methylation: it is mainly caused by the double methylation of 23S rRNA in bacteria. Methylation can reduce the affinity of MLs to the ribosome site to one hundred thousand times of the previous, and is completed by erm enzymes (Farrell et al., 2003). 2. rRNA mutation: such as A2058U/A2058G in 23S rRNA in Streptococcus pneumoniae or A2064G/A2064G in 23S rRNA in Mycoplasma pneumonia (Farrell et al., 2004; Lu et al., 2020). 3. Efflux mediated by mef: mef encodes an efflux pump, which can use MLs as a substrate to expel drugs from the bacteria by consuming energy, thereby reducing the sensitivity of bacteria to drugs (Farrell et al., 2003). 4. Ribosomal protein variants: the most important ones are L4 and L22 (Farrell et al., 2004).

In China, the resistance of many bacteria to MLs is on the rise. Here, we review the situation of drug resistance in China in the past 20-30 years. Search strategy: We searched PubMed, Wanfang database, Zhiwang of China, Google Scholar for articles published before 31 December 2022, by use of the terms: “*Streptococcus pneumoniae*”, “*Mycoplasma pneumoniae*”, “*Bordetella pertussis*”, “*Staphylococcus aureus*”, *“ Group A Streptococcus/Streptococcus pyogenes”, “ Group B Streptococcus/Streptococcus agalactiae,”* AND “macrolide” AND “resistance”, and reference lists of identified studies. Only articles written in Chinese and English were included. Finally, only the most relevant papers for this review were cited. The characteristics of the included literatures are shown in the **Supplementary Table 1**.

## Streptococcus pneumoniae

SP is the most important pathogen in otitis, sinusitis, bronchitis, and community-acquired pneumonia (CAP), as well as a predominant cause of meningitis and bacteremia. The widespread use of MLs is associated with increased resistance. Reports of SP resistance to MLs first appeared in the 1980s–1990s globally and the first report of macrolides-resistant SP (MRSP) in China was also in 1980s (Ye et al., 1988).A total of 295 invasive SP strains were identified from 18 provinces of China during 1981-1983 in that study. Only one of them was resistant to erythromycin (ERY), with a resistance rate of 0.34% (1/295). Since then, domestic studies have found a significant increase in prevalence of MRSP. Li (Li et al., 1999) found that resistance to ERY of SP increased from 2% in the mid-1980s to 79% in the mid-1990s, and clarithromycin (CLA) from 2% in the mid-1980s to 76% in the mid-1990s. In 1997 (Yu et al., 2000), 76.8% of SP were found to be resistant to ERY and children who had taken the drug in the last month were more likely to carry ERY-resistant strains (39 vs 27%, RR 1.5, 95% CI 1.1-2.0). Then, the continuous monitoring in 1998-2000 showed that the ERY resistance rates of the SP in Beijing were 82.2% and 87.4%, respectively (Yu et al., 2001). In addition to Beijing, the increasing resistance rate were also reported in Shanghai (Zhang et al., 2000). The data from Europe for 1992-1998 showed that more than 40% of isolates from France and Italy were resistant to MLs, whereas less than 10% of isolates from Germany, the Netherlands, the Czech Republic, and Poland (Schito et al., 2000), which was lower than China in the same period.

At the turn of the century, Yang (Yang et al., 2002b) carried out drug sensitivity test of SP isolated from nasopharyngeal specimens of children with upper respiratory tract infection in Beijing, Shanghai, Guangzhou and Xi’an. They reported that the resistant rate of 624 isolates to ERY ranged from 75.4% to 96.7%, and 80-99% of the resistant isolates had minimum inhibitory concentration (MIC) ≥256μg/ml. At the same period, they also (Li et al., 2013) reported that 119 of 120 (99.2%) SP with serotype 19F showed resistant to ERY. Of the 119 ERY non-susceptible pneumococci, *ermB* and *mefA* were detected in 115 (96.6%) and 64 (53.8%) isolates, respectively. Both *ermB* and *mefA* were detected in 60 (50.4%) strains. Even more frustrating, 96.6% strains had MIC >256μg/ml. Subsequently, a large number of single and multi-center studies showed that the resistance rate of SP to MLs was in the range of 79%-100% (Liu et al., 2008; Wang et al., 2008; Zheng et al., 2009; Chen et al., 2010b; Xue et al., 2010; Xiong et al., 2012; Zhang et al., 2013c). Only a few single-center studies (Li et al., 2012; Jiang et al., 2013) have shown low rates of resistance to ERY (20%-21.7%). And the resistance rate was lower in adults (69.2%-73.3%) than in children over the same period (Li et al., 2003; Yao et al., 2005; Yang et al., 2008). The Prospective Resistant Organism Tracking and Epidemiology for the Ketolide Telithromycin (PROTEKT) data (1993-2003) also showed a global increase in Mls resistance of SP, from 31% in 1999 to 36.3% in 2003 (Schito and Felmingham, 2005). But, Felmingham collected 20,142 SP isolated from 151 centers in 40 countries between 2001 and 2004, and found there that was no significant temporal trend in the prevalence of MLs resistance in any country. The highest rates (~80%) were recorded among isolates collected in the Far East, followed by South Africa (~54%) and Southern Europe (~37%), whereas resistance was lowest in Latin America (~15%), Australia (~18%) and Northern Europe (~18%). And, telithromycin exhibited good antibacterial activity against SP. The most common macrolide resistance genotype among SP was *erm*(B) (~58%), followed by *mef*(A) (~30%). (Felmingham et al., 2007).

Since 2010, the resistance of MLs has increased even more, with many studies reporting a resistance rate of>92% (Zhao et al., 2017; Fu et al., 2018; Du et al., 2021; Liang et al., 2021; Liu et al., 2021b). One recent study (Zhou et al., 2021) even showed that more than 90% of the strains were resistant to azithromycin (AZM), CLA, and ERY. It is worth noting that MIC_50_ and MIC_90_ of AZM, CLA, and ERY were all>1024μg/ml. Only Shenzhen reported a slight downward trend in SP resistance to MLs (Li et al., 2019c). During this period of time, there was no significant difference in drug resistance between invasive SP and non-invasive SP strains, that is to say, both invasive and non-invasive strains showed high resistance to MLs (Huang et al., 2015b; Lyu et al., 2016; Wang et al., 2019a). Meanwhile, data from China Antimicrobial Surveillance Network (CHINET) (CHINET, 2023) since 2006 showed that both penicillin-sensitive and non-sensitive non-meningitis SP show high resistance rate to MLs (80.2%-100%). Furthermore, 143 of the 144 (99.3%) serotype 14 SP isolates resistant to ERY and the *ermB* gene was determined in all ERY resistant isolates (100%), and the *mefA* gene was positive additionally in 13 of them (9.03%) (He et al., 2015). Meanwhile, studies around the world have showed that macrolide resistance among SP is geographically variable but ranges from <10% to >90% of isolates (Pan et al., 2015; Xiao et al., 2015).

In a word, the onset time of macrolide resistance in SP is basically the same as that in other countries in China. In the past 30 years, the resistance of SP to MLs in children in China has shown an increasing trend, especially in the 1990s, when a sharp upward trend occurred, and then maintained at a high level. *ermB* and *mefA* genes are major SP of resistant to MLs.

## Staphylococcus aureus

SA is an aerobic or facultative anaerobic gram-positive coccus. With strong pathogenicity, it can cause a variety of infections including severe sepsis, pneumonia and wound infection. A growing number of studies have reported that the bacterium is resistant to a variety of antibiotics, especially MLs (Chen et al., 2022).

Resistance to MLs in SA was rarely reported in China before 2000. Huang (Huang and Liu, 1990) compared the resistance rate of SA between 1979-1984 and 1985-1988 in Chongqing, and earlier concluded that the drug resistance rate of ERY increased (P<0.05). Studies in several other southern provinces have also found significant increases in MLs resistance (from 72% to 100% in Guangdong, from 11.1% to 65.8% in Anhui) (P<0.05) (Zhang et al., 1998; Jin et al., 1999; Li and Xiong, 2001). In Beijing, Fan and Ma reported high rates of (79.7-90.3%) MLs resistance (Fan et al., 2000; Ma et al., 2000) and they also found that the resistance rate of methicillin-sensitive SA (MSSA) to ERY was significantly lower than that of MRSA (81% vs 97.1%) (Fan et al., 2000). Meanwhile, data from Guangdong showed that all the 58 MRSA isolated from 1990 to 1995 were resistant to ERY (Zhou, 1997). Prior to 2000, only SA from newborns in Shanxi province in 1995-1997 was reported to have a resistance rate of less than 50% to ERY (Guo et al., 2000). Many reports based on blood samples found that the resistance to ERY fluctuated between 63% and 76% (Duan et al., 2000; Huang and Chen, 2001; Xu and Shao, 2002). It has been reported that the detection rate of SA in sepsis children decreased from 35.4% in 1991 to 5.3% in 2000 (P<0.005) (Xu and Shao, 2002), but no decrease in the resistance of SA to MLs was found. In a word, it is clear that SA showed a significant upward trend in resistance to MLs before 2000. However, H.Westh studied 718 cases of bacteremia with MRSA, occurring between 1959 and 1988 and found that MRSA resistance to MLs occurred at a stable low level (13%) during the whole observation period and always had high MICs to ERY (Westh et al., 1991).

Since 2000, the number of studies on SA resistance to MLs gradually increased. Wang (Wang et al., 2008) monitored the resistance of 7835 SA isolated from children during 2000 to 2006, and found that the resistance rate of ERY was 66.39% and showed an increasing trend (P<0.001). Two studies bases on respiratory specimens in children showed that the resistance rates of SA to ERY was between 45.6%-58.1% (Wu J. et al., 2013) and 77-86.7% (Xia et al., 2012). Two other studies (Zhao et al., 2012a; Chen et al., 2017) found a decrease in the rate of resistance to ERY in SA (81.9-68.2% and 81.9%-54.7%), but the level of MIC was high (MIC90s ≥ 256ug/ml) (Zhao et al., 2012a). At the same time, some studies reported low resistance rate in some areas. For example, the resistance rate of SA to ERY from children in Hangzhou during 2001-2002 was 37.93% (Hua et al., 2003) and it was only 24.5% in Qinghai during 2009 to 2010 (Long and Wang, 2012). Moreover, the date from Zhejiang province from 2007 to 2010 showed low resistance rates of 11.1%, 20.0% and 25.9% (Sun, 2011). In this decade, there have been more studies to compare the resistance of MRSA and MSSA. Almost all studies have shown that the resistance of MRSA to ERY was significantly higher than that of MSSA, regardless of the type of specimen (Hu and Xia, 2009; Shi and Jian, 2010; Wang et al., 2011b; Wang, 2013; Zhang et al., 2013a). Similar findings were made for AZM resistance (Zhang and Jin, 2013). A deeper study (Wang et al., 2011a) found that the resistance rate of MRSA to ERY was as high as 97.9%, and MIC50 and MIC90 were both higher than 256ug/ml. They also found that MRSAs carrying type IV or V *SCCmec* often showed multiple resistance. In conclusion, from 2000 to 2010, the resistance rate of SA to MLs showed a trend of fluctuating increase and then decreasing. MRSA had a higher resistance rate to MLs than MSSA. In Belgium, Olivier Denis found the resistance of 455 clinical MRSA strains to ERY was 64% (Denis et al., 2004).

Since 2010, the research on the resistance of SA to MLs has been more extensive and in-depth. Continuous monitoring from several regions in China showed that the resistance of SA to MLs was on the rise (Huang et al., 2014; Li et al., 2016; Wu et al., 2019; Bao et al., 2021; Ding and Li, 2022). However, the data from CHINET (CHINET, 2023) showed that the resistance rates of MLs in MSSA and MRSA both showed a fluctuating downward trend. And some studies have shown a wide range of resistance rates, making it not easy to draw the conclusion that the drug resistance rate increases or decreases (Zhai et al., 2016; Chi et al., 2018). During this period, there were many studies on the resistance of MRSA and MSSA to MLs, and most studies showed that the resistance rate of MRSA (73.2%-100%) was significantly higher than that of MSSA (16.3%-66.1%) (Huang et al., 2015a; Li et al., 2016; He et al., 2017; Chi et al., 2018; Min et al., 2019; Zhao et al., 2020; Xiao et al., 2021; Zhou et al., 2022). Hu (Hu et al., 2016) reported that the resistance rate of CA-MRSA to ERY in Shenzhen in 2014 was 81.4% and that of HA-MRSA was 86.7%s. Chen (Chen et al., 2022) reported 60% resistance to ERY in CA-MSSA, 86% in CA-MRSA, 55% in HA-MSSA, and 82% HA-MRSA in 2015-2017. In addition to this, the data from the Chinese Pediatric Infectious Disease Surveillance Program (ISPED) (Fu et al., 2021) showed that the resistance rate of MRSA (n=11128) and MSSA (n=20667) to ERY from 11 children’s hospitals during 2016-2020 was 78.2% and 51.9%, respectively. And the data of CHINET (CHINET, 2023) also showed that the resistance rates of MLs in MSSA ranged from 43.9% to 55.3%, while those in MRSA ranged from 93.4% to 73.2%. Antibiotic Resistance Monitoring in Ocular Microorganisms(ARMOR) reported 59.7% of SA isolates overall were resistant to MLs between 2009-2018 (Bispo et al., 2022), which was lower than China.

There have also been studies of resistance in SA from different sources of samples, but no significant differences have been found. Specimens from skin, soft tissue, pneumonia, pus, etc., all showed high (62.3%-98.5%) drug resistance rates (Deng et al., 2012; Deng et al., 2013; Ning et al., 2014; Zhang et al., 2014a; Ran et al., 2017). Some studies also analyzed resistance rates in both children and adults, but the results are not very clear. For example, a recent study (Li et al., 2020b) found lower rates of ERY resistance in adult than in childhood (36.60% vs 46.31%, P<0.05). However, Ma (Ma et al., 2007) reported that the resistance rate of SA from adults to ERY was 89.8%, while that from children was 49.0%. Two other studies analyzed the causes of MLs resistance. Li (Li et al., 2020b) found that the resistance rate of Class I integron-positive isolates to ERY was higher than that of class I integron-negative isolates (50.85% vs39.29%, P<0.05). Chen (Chen et al., 2014a) reported that the *mecA* positive strain had a higher resistance rate to ERY than that of negative strain (80.30% vs 35.0%).

From the literature above, we can see that the research on the resistance of SA to MLs first experienced a rapid rising and then showed a decreasing trend of volatility, with certain regional differences in China. And, the resistance rate of MRSA was higher than MSSA.

## Group A streptococcus

GAS, alternatively termed *Streptococcus pyogenes*, is a Gram-positive pathogen that routinely causes a variety of non-invasive (pharyngitis, impetigo, cellulitis, etc) and invasive infections (necrotising fasciitis, sepsis, etc). MLs is an important treatment for GAS infections. In the present study, we investigated trends in GAS resistance to MLs in China.

In 1968, a strain of GAS resistant to ERY isolated from a throat swab in a child was reported for the first time in the world (Sanders et al., 1968). In Europe, the ML resistant-GAS strain first appeared in Spain in the 1990s, and despite this, studies in Spain since then have shown that GAS has a resistance rate of 0% to ERY (Ardanuy et al., 2010). The first drug susceptibility test in mainland China was conducted by Su (Su et al., 2003), who collected 620 strains from Guangzhou, Jilin, Hubei and Chongqing during 1988-1994. The resistance rate of GAS to ERY was 35.2%. Later, Dong (Dong et al., 1999) collected strains from various provinces and cities during 1993-1994 and found that the resistance rate of ERY was different in different regions: 75.26% in Jilin, 22.22% in Sichuan, 1.1% in Hubei, and 27.19% in Guangzhou. In addition to differences in drug resistance between different cities, Dong (Dong et al., 2001) found that there were also differences between urban and rural areas: during 7 consecutive years of surveillance, GAS resistance to ERY in rural children in Guangzhou (21.4%-67.4%) was much more severe than that in urban children (4.4%-23.8%). Lin (Lin et al., 2010) reported that the resistance rate of GAS to telithromycin also showed an increasing trend, from 20.37% during 1993-1994 to 87.93% during 2005-2008. Similarly, the resistance rate of Josamycin (JOS) increased from 84.8% in 1993-1994 to 98.1% in 2005-2008 and the CLA resistance rate of GAS in Beijing area during 1993-1994 was 79% (Wu et al., 2014). In a word, the GAS resistance rate of MLs in China before 2000 was relatively high, although there were regional differences.

Deng (Deng et al., 2008) collected 47 strains in children with impetigo from 2005 to 2007 in Beijing, and the resistance rate of ERY was 47.1%. In 2009, a total of 265 GAS strains were collected from 14 hospitals, of which 82.1% were resistant to ERY (Wang et al., 2010). During the same period, many multi-center studies showed that the resistance rate of ERY in Beijing, Shanghai, Liaoning, Sichuan, Shenzhen were all above 90% (Liang et al., 2008; Liu et al., 2009; Chang et al., 2010; Chen et al., 2012; Ji et al., 2012; Zhou et al., 2014a). Meanwhile, the drug resistance rate of AZM in Beijing, Tianjin and other areas were also higher than 95% (Liang et al., 2008; Liu et al., 2009; Chang et al., 2010; Ji et al., 2012; Yin et al., 2018). Other kind of MLs were also at high drug resistance levels. For example, the resistance rates of GAS from Beijing, Shanghai, Chongqing, Tianjin to CLA were all >90% (Liang et al., 2008; Ma et al., 2008; Liu et al., 2009; Chang et al., 2010; Feng et al., 2010a; Ji et al., 2012; Wu et al., 2014; Yin et al., 2018) and the resistance rates of roxithromycin and doxomycin are also ≥90% (Han et al., 2007; Ma et al., 2008; Feng et al., 2010a). In conclusion, the data from 2000-2010 in China show that the resistance rate of GAS to MLs is on the rise, especially after 2005. However, a decline in the rate of resistance to ERY in GAS in several abroad regions was reported after 2005 (<10%) (Ardanuy et al., 2010; Montes et al., 2014; Berbel et al., 2021).

After 2010, the resistance rate of GAS to MLs remained high. Wang (Wang et al., 2013a) reported that 71 strains collected in Beijing in 2011 were all resistant to ERY (100%). Also in Beijing, Zhu (Zhu et al., 2021) collected a total of 234 strains from 2013 to 2019, and the resistance rate of ERY was as high as 98.29%. Sun (Sun et al., 2022) compared the ERY resistance rate of 50 strains in Shenzhen from 2016 to 2020, and it was all>96% in the other years except for 92% in 2017. In 2017, the resistance rate of 66 strains to ERY in a hospital in Guangzhou was as high as 96.97% (Tan et al., 2019). It has been reported that 35 GAS strains cultured from throat swabs of children in Beijing were 100% resistant to mediamycin, and the resistance rate to acetylspiramycin was 97.14% (Yang et al., 2015a). In Asia, in addition to China, Japan also has a high rate of MLs resistance (Tatara et al., 2020; Ikebe et al., 2021).

There are few studies on GAS resistance genes in China. The earliest strains related to GAS resistance genes in mainland China came from 2003 (Chang et al., 2010). Of 91 MLs resistant isolates, 77 (84.6%) had the *ermB* gene, while 14 isolates (15.4%) had the *ermA* gene. Ji (Ji et al., 2012) collected 52 strains from children with impetigo from 2003 to 2008 in Beijing, among which 92.3% carried *ermB* and 7.7% carried *ermA*. In addition, *ermB* genes accounted for more than 90% of the strains from Beijing, Shanghai, Shenzhen, and Guangzhou before 2010 (Deng et al., 2008; Ma et al., 2008; Feng et al., 2010a; Feng et al., 2010b). A study from Shandong after 2010 showed that the *ermB* gene accounted for up to 100% of all strains (Liu et al., 2015).

To sum up, the resistance of GAS to MLs in China shows an obvious upward trend, with some fluctuations, and the resistance varies in different regions. *ErmB* gene is an important gene for GAS resistance to MLs, and it is dominant in China.

## Group B Streptococcus

GBS, also known as *Streptococcus agalactiae*, is a β-hemolytic Gram-positive bacterium that colonizes the lower genital tract as an asymptomatic microbe. However, it can be highly pathogenic if it was established in other host niches. During pregnancy, ascending GBS infection is associated with preterm birth, stillbirth, and fetal injury. In addition, fetuses and neonates are uniquely susceptible to GBS infections, which most commonly include sepsis, pneumonia, meningitis, and encephalopathy. MLs are one of the first-line treatments for GBS. In the past decades, resistance to MLs continue to rise all over the world.

The earliest article on the resistance of GBS to ERY in China was published in 1989 (Cao et al., 1989). They obtained vaginal secretions of pregnant women and skin wipes of newborns from Beijing. A total of 10 strains of GBS were cultured and no ERY resistant strains was found. Only 2 years later, Zhang (Zhang et al., 1995) reported 53 strains also from pregnant women and newborns, and the resistance rate to ERY was as high as 66.04%. However, a susceptibility test of the GBS strain in Australian in 1999 showed that the ERY resistance rate was only 2.8% (Stylianopoulos et al., 2002). Shen (Shen et al., 2005) reported that the resistance rate of GBS to ERY was 8% in Beijing in 1998, increased to 16% in 1999, and 45% in Guangzhou in 1999, which was significantly higher than that in Beijing (p<0.01). Of the 45 ERY-resistant isolates, 44% contained *ermB* and 29% contained *mefA*, 13.33% contained both *ermB* and *mefA* genes. Yang (Yang et al., 2002a) compared the resistance of 113 GBS from Beijing (n=69) and Guangzhou (n=31), China, and St. Petersburg (n=13), Russia between 1996 and 1999. Forty-six percent of the isolates from China were ERY resistant while the isolates from Russia were all susceptible. The *ermA* gene was detected only in Beijing strain. Thirty-four strains (30.1%) carried *ermA* and/or *ermB*, of which 33 (97.1%) strains were resistant to ERY, no *ermC* gene was found. However, 53 strains carrying *mreA*, 18 strains carrying *mefA*, and 1 *ermA* positive strain were still sensitive to ERY. Two resistant strains did not carry any detected genes (*mreA*, *mefA* and *ermA/B/C*). In Canada, a study of 32 ERY-resistant GBS strains were genotyped and 88% were found to be *erm* positive (54% constitutive) and 12% *mef* positive (De Azavedo et al., 2001).

It was reported that only 8.6% of 46 strains of GBS isolated from maternal cervical secretions and neonatal pharyngeal during 2003-2006 were resistant to ERY (Zhao et al., 2007). Among 193 strains isolated from throat swabs of children and vaginal secretions of pregnant women in Jingdezhen between 2004 and 2008, the ERY resistance was also only 15% (Lin et al., 2015). However, Guo (Guo et al., 2012) reported that the resistance rate of ERY in Zhejiang Province was as high as 86.2% in 2006, 86.1% in 2007, and 84.8% in 2010. A number of studies (Guo et al., 2012; Li et al., 2015; Lin et al., 2015; Zhang et al., 2015b; Lin et al., 2022a) comparing the changes in GBS resistance rates in different years suggested that the high ERY resistance rate in the 10 years from 2001 to 2010, most of which fluctuated between 41% and 93.3%. The study (Chen et al., 2010a) from Guangzhou also showed that *ermB* gene expression accounted for 71.1%, *mefA* 52.2% and *mefE* 68.9% in ERY resistant strains.

There has been an increase in research on GBS resistance since 2010. Studies in children mainly focused on invasive infections in neonates, and most of these studies have been based solely on blood specimen (Wu X. et al., 2013; Chen et al., 2014b; Huang et al., 2018; Wang et al., 2018; Yu and Hu, 2018; Zhan et al., 2018; Liang and Wang, 2019; Liu et al., 2019; Xie and Liu, 2020; Liu et al., 2021a; Lin et al., 2022a; Qu et al., 2022), with a few studies on other specimens, such as cerebrospinal fluid (CSF) and sterile body fluid (Huang et al., 2016; Li et al., 2019a). These studies have shown that the rate of ERY resistance among invasive GBS strains has fluctuated between 43.33% and 100% (Wu X. et al., 2013; Chen et al., 2014b; Huang et al., 2018; Wang et al., 2018; Yu and Hu, 2018; Zhan et al., 2018; Liang and Wang, 2019; Liu et al., 2019; Xie and Liu, 2020; Liu et al., 2021a; Lin et al., 2022a; Qu et al., 2022), among which only two studies (Huang et al., 2018; Liang and Wang, 2019) from Shanghai and Zhejiang province showed that GBS resistance to ERY was less than 50%. A multi-center continuous monitoring in southern China (Li et al., 2019a; Li et al., 2020a) showed a fluctuating upward trend in the resistance rate of ERY (66.7 in 2013 to 78.6% in 2016). They also found that 48 of 56 (85.7%) ERY resistant strains carried the *ermB* gene and two (3.6%) strains carried both *ermB* and *mefA*. However, it should be noted that 100% of the 17 intermediate strains carried *ermB* and 5.9% also carried *mefA*. In the United States, ERY resistance rates are also high in the GBS strains that cause invasive adult and neonatal disease, at 54.8% and 44.8%, respectively (Francois Watkins et al., 2019; Nanduri et al., 2019).

In addition to neonates, studies (Lei et al., 2015; Wang et al., 2015a; Zhang et al., 2015a; Zhong et al., 2015; Li et al., 2018c; Lin et al., 2019; Wang et al., 2019b; Li et al., 2020a) on the resistance of GBS strains isolated from infants and young children were also mainly derived from invasive infection strains. Among these studies, only one study (Zhang et al., 2015a) from Shenzhen reported that the resistance rate of ERY was 41% and the intermediate rate was 25%. Other studies all showed that the resistance rate of ERY was higher than 75%, the highest was 92.5% (Wang et al., 2015b) and MIC50-90 was higher than 256μg/ml. Similarly, most AZM resistance rates have been above 95% since 2010 (Wang et al., 2015a; Wang et al., 2015b; Wang et al., 2020). The earliest study on the resistance rate of CLA can be traced back to 2008. Wang (Wang et al., 2015a) collected 40 strains in Shenzhen and Beijing from 2008 to 2013 and found that 92.5% of the strains resistant to CLA. So far, studies on the CLA resistance rate of GBS from different regions in China are all lower than this value, but the overall resistance rate is still high. For example, Zhang (Zhang et al., 2018) reported that the resistance rate of GBS to CLA in Kunming, Yunnan Province was 42.5%, which is the lowest reported level in China. The study on the resistance rate of GBS to telithromycin was relatively late and rare. It was reported that the resistance rate of 40 strains during 2008-2013 was 0 (Zhong et al., 2015), and 48 strains isolated during 2013-2014 was 30.56% (Wang et al., 2015a). Studies on the detection of resistance genes increased during this decade, with the *ermB* gene accounting for more than 80% in Guangzhou, Beijing, Shanghai and Jiangxi province (Li et al., 2018a; Li et al., 2018b; Nie et al., 2018; Li et al., 2019a; Du, 2022). In Sub-Saharan Africa, a meta analysis shows the resistance rate of ERY was only 25.11% (Wadilo et al., 2023).

In conclusion, the resistance rate of GBS to MLs has remained at a high level in China, with the *ermB* gene dominant.

## Bordetella pertussis

BP is the responsible pathogen of pertussis, an acute respiratory infectious disease that occurs in children usually and causes paroxysmal, spasmodic cough. MLs represented by ERY have been the first choice for the prevention and treatment of pertussis. The first ML-resistant BP (MRBP) strain was reported in 1994 in a 2-month-old infant in Yuma, Arizona, US (Lewis et al., 1995). Since then, countries such as the United Kingdom, France, Iran and even East Asian and Southeast Asian countries including Japan, Vietnam and Cambodia, have reported the appearance of MRBP, but the prevalence in these countries is generally low (0.5% to 18.2%) (Korgenski and Daly, 1997; Wilson et al., 2002; Bartkus et al., 2003; Guillot et al., 2012; Shahcheraghi et al., 2014; Kamachi et al., 2020; Yamaguchi et al., 2020; Koide et al., 2022).

The earliest study on the drug resistance of BP in mainland of China was published in Jun 2008 (Ou, et al, 2008). In this study, 16 strains of BP were collected from 2000 to 2007 in Beijing and 4 strains were from 1970s. The results showed that all strains were ML-sensitive, and the MICs of ERY, AZM and CLA were very low. There were few studies on the resistance of BP in the following period in China, but combining the research conducted abroad of the same period (Korgenski and Daly, 1997; Wilson et al., 2002; Bartkus et al., 2003; Guillot et al., 2012);, we concluded that the incidence of ML-resistant BP was low in this period globally.

Then, in 2011, a cross-sectional study of BP seropositivity and carriage among healthy adolescents in Shandong Province, 2 ERY-resistant strains were identified with MIC>256μg/ml, and both clinical isolates had the A2047G mutation in the 23S rRNA (Zhang et al., 2013b). In 2012, 4 strains of ML-resistant BP were found in Xi’an, with A2047G mutation and all MICs of ERY, CLA and AZI were all >256ug/mL (Wang et al., 2013b). Several other studies have also shown that the molecular mechanism of ERY resistance is mainly related to the A2047G mutation in the V domain of 23S rRNA (Guillot et al., 2012; Wang et al., 2013b). A study in Xi’an (Wang et al., 2014) included 16 strains from 2012-2013, of which 87.5% (14/16) were resistant to ERY and all had A2047G mutation. Meanwhile, of the 100 samples positive for 23S rRNA PCR, 85 (85.0%) were found to have the A2047G mutation by sequencing. About the same time, data from Beijing showed that 91.9% (91/99) strains were resistant to MLs (MIC>256μg/ml), and all but one ERY-resistant strain contained the A2047G mutation (Yang et al., 2015b). Further studies conducted in Xi’an showed that the drug-resistant pertussis rate in Xi’an was 75.86-100% during 2012 to 2020, and the MIC50s of the involved bacteria to ERY, AZI and CLA were all >256μg/ml. However, some studies from south China such as Guangdong, Zhejiang, Hunan and Shanghai showed a relatively lower resistance rate (48.6%-77.1%) (Hua et al., 2019; Yan, 2019; Zhe et al., 2019; Zhang et al., 2020; Lin et al., 2022b), while it was generally higher in northern China (79.3%-100%) (Wang et al., 2014; Li et al., 2018; Li et al., 2019b; Li et al., 2019d; Juan et al., 2022).

Due to the high difficulty of pertussis culture, many regions do not carry out pertussis culture and susceptibility tests. Therefore, there is relatively little literature on pertussis resistance in China. Nevertheless, from the few studies at present, we can still see that the probability of BP resistant to MLs has increased significantly since 2010, although there are some regional differences. In order to overcome the difficulty of BP culture and avoid the missed diagnosis of drug-resistant BP, some studies also recommend the use of PCR-based sequencing for rapid detection of possible antimicrobial resistance (Wang et al., 2014; Zhang et al., 2017). Given that pertussis is clearly on the rise in China, concern about pertussis resistance is an ongoing and important issue.

## Mycoplasma pneumoniae

MP is one of the main pathogens of CAP in children and adolescents. MLs are the main drugs for the treatment of MP infection. From 1968 to 1999, ML-resistant MP (MRMP) has been reported in only a few countries (Niitu et al., 1970; Stopler and Branski, 1986; Critchley et al., 2002; Pereyre et al., 2007). However, since 2000, the resistance rate has increased significantly and continuously (Okazaki et al., 2001; Morozumi et al., 2008).

The detection of drug resistance of MP started late in China but high rates of resistance were found at the outset. It was not until 2003 that Chinese scholars first reported that MP was 100% sensitive to AZM and roxithromycin (ROX), but all of them were resistant to ERY (Guo and Mai, 2003). To our knowledge, this is the first report of MRMP in China. In 2005, Xin reported (Xin et al., 2005) that 80% (4/5) of MP isolated in Beijing from 2003 to 2004 were resistant to ERY, ROX, CLA and AZM. Subsequently, they continued reported (Xin et al., 2006; Xin et al., 2009) the resistance rate of MLs was 69.2% (9/13) to 92% (46/50). The MICs of ERY, AZM and JOS were 512->1024μg/ml, 16-64 and 8-64 µg/mL, respectively. Meanwhile, they found all the drug-resistant strains were accompanied by 23S rRNA mutations, including A2063G (80%), A2063C (2%) and A2064G (10%) (Xin et al., 2009). Chen reported that the resistance rate to ERY in Hangzhou from 2006 to 2008 was 54.5-60.7% and it reached 78.9% (15/19) in 2009 (Chen et al., 2009). In a word, the MLs resistance of MP in China discovered late, but showed high resistance as soon as it was discovered.

Studies from 2010 onwards also showed high levels of resistance. Many studies showed that the resistance rate of MP to ERY in Beijing is relatively high and most of which were above 80% (Dong et al., 2013; Tian et al., 2013; Yin et al., 2013). Even in the study of 235 strains showed that the resistance rate of ERY reached 87% (Zhou et al., 2014b) in 2014. Similar trend was also found in other cities in Zhejiang province, and the drug resistance rate was relatively high (70.59%-100%) (Zhou et al., 2015; Chen et al., 2018; Lin et al., 2021). The study conducted in Guangdong showed that the resistance rate of ERY was 66.47-69.46% during 2014 to 2018. It’s relatively more sensitive to AZM (9.79%), ERY (5.34%), erythromycin istolate (1.59%), ROX (3.13%) and CLA (2.99%), but the resistance rate of JOS(36.44%) and acetylspiramycin (48.17%) to MP is higher (Chen et al., 2019). In subsequent reports, the resistant rate has gradually increased, fluctuating between 24% and 77% (Lu et al., 2020; Hung et al., 2021). According to the studies in other provinces and regions, the overall trend of MLs resistant MP in China is on the rise, but the reported resistance rate in Guangdong (5.34%), Yunnan (1.08%-5.64%) and Shanxi (64%) is lower than that in other regions (Ye et al., 2013; Chen et al., 2019; Jia et al., 2022; Lin et al., 2022c). In addition, some other studies conducted in Guangdong also showed that the rate of ERY in Guangdong (42.15-63%) was lower than that in Tianjin(92%), Shanghai (85.7%). (Liu et al., 2010; Xu et al., 2013; Ma et al., 2014; Du et al., 2017; Du et al., 2020). The distribution of MRMP is not even in the world. The resistance rate of MLs in East Asian countries represented by China is relatively high, while that in European and American countries is relatively low. Since the beginning of reporting, the drug resistance rate in Japan has shown an upward and then downward trend, where in 2009-2011, the rate was the highest at 72%. The reporting rate of European and American countries is relatively low, ranging from 0 to 27% (Wang et al., 2022).

Previous studies have revealed that there are 2 types of clinical isolates (type 1 and type 2), differing significantly in their P1 gene sequences (Su et al., 1990), and the resistance rate of type 2 isolates is relatively low (Zhao et al., 2012b; Xue et al., 2014; Shi et al., 2017; Qu et al., 2019). Before 2012, the epidemic strain in Beijing was type 1 for the P1 gene, and the genotype shift from type 1 to type 2 began in 2013 (Waites et al., 2008), which may explain the decrease in the proportion of MRMP in recent studies in Beijing. Several studies have shown that the A2063G mutation in the 23S rRNA gene domain V is the most common in MRMP isolates in China. In addition, A2064G, A2063C/T, C2617G/A, A430G, T279C, T508C, etc. are associated with the resistance (Zhang et al., 2014b; Jiang et al., 2021). A study in Zhejiang showed that (Zhou et al., 2015), A2063G channels are responsible for the inhibition of 14- and 15-member ring MLs (ERY MIC: 128->256μg/ml and AZM MIC: 32->64μg/ml, etc.), while retaining activity against 16-member ring MLs (JOS 1-8μg/ml).

From the current study, it can be seen that the incidence of MRMP is increasingly high in China, and only relatively low in a few areas. Although the drug resistance of MP cannot enhance the virulence of the pathogen, it will increase the difficulty of clinical treatment and limit the choice of antibiotics, and will cause more complications if the treatment is not timely (Zhou et al., 2014b). Therefore, it is necessary to continue to promote the monitoring of resistance.

## Conclusion

In conclusion, various common pathogens such as SP, GAS, GBS, SA, BP and MP, have shown high resistance rates and high resistance level to MLs in Chinese children. The drug resistance of some pathogens occurs suddenly (such as MP and BP), which is a high drug resistance rate and high drug resistance level. There are regional differences in the resistance level of these pathogens. Mutations in the 23S rRNA site and/or carrying the *ermB* gene are currently common causes of resistance in these pathogens.

## Author contributions

XM and LL contributed to conception and design of the study. JL, JG, HZ, XW and MX organized the database. LL and XM wrote the first draft of the manuscript. JL, HZ and JG wrote sections of the manuscript. All authors contributed to manuscript revision, read, and approved the submitted version.
