# Supplementary material for: Severe problem of macrolides resistance to common pathogens in China
Source: Front Cell Infect Microbiol. 2023 Aug 10;13:1181633. doi: 10.3389/fcimb.2023.1181633 (PMC10448830; doi:10.3389/fcimb.2023.1181633)
Supplement: Supplementary file 1 [file Table_1.docx]

| Supplementary Table 1. Trends of *Streptococcus pneumoniae* resistance to macrolide in Chinese children. | | | | | | | | | | |  | |  |
| --- | --- | --- | --- | --- | --- | --- | --- | --- | --- | --- | --- | --- | --- |
| Authors | Number of strains (n) | Period (year) | Type of specimens | District | Resistance to erythromycin | | | Resistance to azithromycin | | | Resistance gene | | References |
|  |  |  |  |  | Resistance rate(%) | MIC 50(ug/ml) | MIC90(ug/ml) | Resistance rate(%) | MIC 50(ug/ml) | MIC90(ug/ml) | *Erm*B  (%) | *mefA/E*  (%) |  |
| Ye et al. | 295 | 1981-83 | CSF, Blood, MEF | Throughout the country | 0.34 | -- | -- | -- | -- | -- | -- | -- | Ye et al., 1988 |
| Li et al. | 49  33 | 1983-85  1995-97 | CSF, blood, MEF and pharyngeal secretion | Beijing | 2  79 | -- | -- | -- | -- | -- | -- | -- | Li et al., 1999 |
| Yu et al. | 190 | 1997.11-12 | NPS | Beijing | 76.8 | -- | -- | -- | -- | -- | -- | -- | Yu et al., 2000 |
| Yu et al. | 421 | 1997-982000 | NPS | Beijing | 81.0 | -- | -- | -- | -- | -- | -- | -- | Yu et al., 2001 |
| Zhang et al. | 90 | 1999 | sputum | Shanghai | 73.3 | -- | -- | -- | -- | -- | -- | -- | Zhang et al., 2000 |
| Li et al. | 120 | 1997-06  2010 | NPS | Beijing | 100 | 256 | 256 | -- | -- | -- | 94.2 | 56.7 | Li et al., 2013 |
| He et al. | 144 | 1997-12 | sputum, [nasopharyngeal](javascript:;)secretions | Beijing, Shanghai, Guangdong, Chongqing, Shanxi | 99.3 | >256 | >256 | -- | -- | -- | 100 | 9.1 | He et al., 2015 |
| Yang et al. | 654 | 2000-01 | NPS | Beijing, Shanghai, Guangdong, Xi’an | 84.7 | >256 | -- | -- | -- | -- | -- | -- | Yang et al., 2002b |
| Li et al. | 30 | 2000-01 | sputum, blood, urine, pus, CSF, [PE](javascript:;) | Haerbin, Beijing, Dalian, Tianjin, Nanjing,Shanghai, Hangzhou,Wuhan | 73.3 | -- | -- | -- | -- | -- | -- | -- | Li et al., 2003 |
| Yao et al. | 892 | 2000-02 | NPS | Beijing Shanghai Guangzhou | 84.3 | 256 | 512 | -- | -- | -- | -- | -- | Yao et al., 2005 |
| Wang et al. | 4781 | 2000-06 | sputum, blood, CSF, urine, pus, NPS | Beijing Shanghai Guangzhou Chongqing | 90.8 | -- | -- | -- | -- | -- | -- | -- | Wang et al., 2008 |
| Yang et al. | Adult：39  Child：122 | 2004-05 | sputum | Shanghai | Adult: 69.2  Child：94.3 | Adult：32  Child：>32 | Adult：>32  Child：>32 | -- | -- | -- | 52.6 | 5.2  *mefE* | Yang et al., 2008 |
| Liu et al. | 451 | 2005-06 | sputum, blood, throat swab, PE | Beijing,Shanghai,Shenzhen,Chengdu,Nanjing,Wuhan,Shenyang,Hangzhou | 95.1 | >256 | >256 | -- | -- | -- | -- | -- | Liu et al., 2008 |
| Xue et al. | 171 | 2006-08 | NPS | Throughout the country | 95.9 | >256 | >256 | -- | -- | -- | -- | -- | Xue et al., 2010 |
| Chen et al. | 31 | 2007 | sputum | Suzhou | 100 | -- | -- | -- | -- | -- | -- | -- | Chen et al., 2010b |
| Zheng et al. | 62 | 2007-08 | sputum, throat swab, blood, CSF, pus | Fujian | 79 | -- | -- | -- | -- | -- | -- | -- | Zheng et al., 2009 |
| Zhang et al. | 39 | 2007-11 | sputum, blood, CSF | Throughout the country | 89.7 | -- | -- | -- | -- | -- | -- | -- | Zhang et al., 2013c |
| Jiang et al. | 15 | 2008-12 | CSF | Yunnan | 20 | -- | -- | -- | -- | -- | -- | -- | Jiang et al., 2013 |
| Li et al. | 121 | 2007-10 | sputum | Shandong | 21.7 | -- | -- | -- | -- | -- | -- | -- | Li et al., 2012 |
| Xiong et al. | 45 | 2009-10 | blood, CSF, PE | Shenyang | 100 | -- | -- | -- | -- | -- | -- | -- | Xiong et al., 2012 |
| Zhou et al. | 300 | 2010-15 | blood, CSF, PE | Beijing | 96 | >1024 | >1024 | 96 | >1024 | >1024 |  |  | Zhou et al., 2021 |
| Huang et al. | Invasive: 23  Non-invasive: 71  <5 years：42  >51 years:44 | 2011-13 | Invasive：blood, CSF, PE  Non-invasive：sputum, tracheal/bronchial aspirates and ear secretions. | Guangdong | Invasive：87  Non-invasive:78.9  <5years：96  >51years：75 | -- | -- | -- | -- | -- | -- | -- | Huang et al., 2015b |
| Zhao C[25] | 881 | 2011-16 | sputum, blood, BALF, throat swab, CSF | Throughout the country | 95.2 | -- | > 256 | 96.9 | -- | > 256 | -- | -- | Zhao et al., 2017 |
| Lyu et al. | Invasive:21  Non-invasive: 166 | 2013-14 | Invasive(Blood，CSF，PE) Non-invasive(sputum、BALF、NPS | Beijing | Invasive：100  Non-invasive：99.4 | Invasive：256  Non-invasive：256 | Invasive：256  Non-invasive：256 | -- | -- | -- | -- | -- | Lyu et al., 2016 |
| Liang et al. | 419 | 2015-17 | sputum, blood, BALF | Guangdong,Guangxi,Hunan | 94.3 | -- | -- | -- | -- | -- | -- | -- | Liang et al., 2021 |
| Li et al. | 86 | 2013-18 | sputum, BALF | Shenzhen | 27.91% | -- | -- | -- | -- | -- | -- | -- | Li et al., 2019c |
| Wang et al. | Invasive：165  Non-invasive 5967 | 2016 | sputum, BALF, nasal/TS nasopharyngeal/tracheal aspirate high vaginal/eye/ear swab, PE, pus, urine, ascites, synovial fluid, aseptic humoral, blood, CSF | Throughout the country | Invasive：95.2  non-invasive：97.1 | -- | -- | -- | -- | -- | -- | -- | Wang et al., 2019a |
| Fu et al. | 8354 | 2017 | sputum, urine, feces, CSF, TS | Throughout the country | 97.8 | -- | -- | -- | -- | -- | -- | -- | Fu et al., 2018 |
| Du et al. | 387 | 2018-20 | sputum，blood，wound secretions | Sichuan | 94.4 | -- | -- | 80.6 | -- | -- | -- | -- | Du et al., 2021 |
| Liu et al. | 809 | 2018-20 | sputum, blood, urine, pus, CSF | Sichuan,Ningxia,Xinjiang,Guangdong | 96.3 | -- | -- | -- | -- | -- | -- | -- | Liu et al., 2021b |

Note: PE- [pleural effusion](javascript:;), CSF-cerebrospinal fluid, BALF- bronchoalveolar lavage fluid,MEF- middle ear fluid, PE-pleural effusion, Nasopharyngeal secretions--NPS ,TS- throat swab

Supplementary Table 2. Trends of *Mycoplasma pneumoniae* resistance to macrolide in Chinese children.

| Authors | Number  (n) | Period  (year) | Specimen | District | Resistance of erythromycin | | | Resistance of azithromycin | | | Resistance of clarithromycin | | | Resistance of roxithromycin | | | Resistance of josamycin | | | Mutation site | | | References |
| --- | --- | --- | --- | --- | --- | --- | --- | --- | --- | --- | --- | --- | --- | --- | --- | --- | --- | --- | --- | --- | --- | --- | --- |
|  |  |  |  |  | Resistant rate(%) | MIC 50  (ug/ml) | MIC90  (ug/ml) | Resistant rate(%) | MIC 50  (ug/ml) | MIC90  (ug/ml) | Resistant rate(%) | MIC50  (ug/ml) | MIC90  (ug/ml) | Resistant rate(%) | MIC 50  (ug/ml) | MIC90  (ug/ml) | Resistant rate(%) | MIC 50  (ug/ml) | MIC90  (ug/ml) | 2063 | 2064 | other |  |
| Xin et al. | 5 | 2003-04 | PS | Beijing | 80 | 100 | 100 | 80 | 2000 | 2000 | 80 | 200 | 2000 | 80 | 200 | 2000 |  |  |  |  |  |  | Xin et al., 2005 |
| Xin et al. | 50 | 2003-06 | PS | Beijing | 92 | 128 | 512 | 92 | 128 | 256 |  |  |  |  |  |  | 92 | 32 | 64 | 1. G 80 2. A-C 2 | A-G 10 |  | Xin et al., 2006 |
| Xin et al. | 13 | 2004-05 | PS | Beijing | 69.2 | 128 | ＞1024 | 69.2 | 16 | 64 |  |  |  |  |  |  | 69.2 | 8 | 64 |  |  |  | Xin et al., 2009 |
| Chen et al. | 19 | 2006 | PS |  | 78.9 | ＞256 | ＞256 | 78.9 | 32 | 64 | 78.9 | ＞256 | ＞256 | 78.9% | ＞256 | ＞256 | 78.9 | 16 | 64 | A-G 78.9 |  |  | Chen et al., 2009 |
| Liu et al. | 100 | 2008-09 | PS | Shanghai | 90 | ＞128 | ＞128 | 90 | ＞128 | ＞128 | 90 | ＞128 | ＞128 |  |  |  | 90 | 4 | 8 | A-G 88  A-T 2 | A-G 2 |  | Liu et al., 2010 |
| Ye et al. | - | 2008-10 | PS | Shanxi |  |  |  |  |  |  |  |  |  |  |  |  |  |  |  | A-G 24 | A-G 6 | A2067G 2 | Ye et al., 2013 |
| Zhou et al. | - | 2009-10 | PS, sputum | Zhejiang |  |  |  |  |  |  |  |  |  |  |  |  |  |  |  | A-G 84.68  A-T 2.55 | A-G 0.43 |  | Zhou et al., 2014b |
| Dong et al. | 30 | 2010 | PS | Beijing | 100 | 128-512 | |  |  |  |  |  |  |  |  |  |  |  |  | A-G 100 |  |  | Dong et al., 2013 |
| Tian et al. | 40 | 2010 | PS | Beijing | 100 | 64-256 | |  |  |  |  |  |  |  |  |  |  |  |  |  |  |  | Tian et al., 2013 |
| Ma et al. |  | 2010-11 |  | Guangdong |  |  |  |  |  |  |  |  |  |  |  |  |  |  |  | A-G 63 |  |  | Ma et al., 2014 |
| Xu et al. |  | 2010-11 | BALF | Tianjin |  |  |  |  |  |  |  |  |  |  |  |  |  |  |  | A-G 92 |  |  | Xu et al., 2013 |
| Yin et al. | 53 |  | PS | Beijing | 71.7 |  |  | 60.4% |  |  |  |  |  |  |  |  |  |  |  |  |  |  | Yin et al., 2013 |
| Zhang et al. | 115 | 2011-13 | PS | Guangdong | 60.1 |  |  | 28.06 |  |  | 33.89 |  |  | 68.53 |  |  |  |  |  |  |  |  | Zhang et al., 2014b |
| Chen et al. | - | 2011-16 | sputum | Zhejiang |  |  |  |  |  |  |  |  |  |  |  |  |  |  |  | A-G 52.2 |  |  | Chen et al., 2018 |
| Zhou et al. | 71 | 2012-14 | PS | Zhejiang | 100 | 128-＞256 | | 100 | 32-＞64 | | 100 | 128-＞256 | | 100 | 0.064-1 | | 100 | 1-8 | | A-G 100 |  |  | Zhou et al., 2015 |
| Chen et al. | 10065 | 2014-18 | PS, sputum | Guangdong | 5.34 |  |  | 9.79 |  |  | 2.99 |  |  | 3.13 |  |  | 36.44 |  |  |  |  |  | Chen et al., 2019 |
| Wang et al. | - | 2015-20 |  | Beijing |  |  |  |  |  |  |  |  |  |  |  |  |  |  |  | A-G 45.96  A-C 0.23 | A-G 0.35 |  | Wang et al., 2022 |
| Jia et al. | 185 | 2015-20 | sputum |  | 1.08 |  |  | 8.65 |  |  | 4.86 |  |  | 4.86 |  |  | 10.27 |  |  |  |  |  | Jia et al., 2022 |
| Du et al. | 102 | 2016 | PS | Guangdong | 42.15 |  |  | 66.67 |  |  | 59.8 |  |  | 54.9 |  |  | 68.63 |  |  |  |  |  | Du et al., 2017 |
| Zhao et al. | 154 | 2017-18 | PS | Jilin, Beijing, Shandong, Anhui, Jiangsu | 79.9% | 128-＞256 | | 79.9% | 2-32 | |  |  |  |  |  |  |  |  |  |  |  |  | Zhao et al., 2019 |
| Lin et al. | - | 2017-19 | PS | Zhejiang |  |  |  |  |  |  |  |  |  |  |  |  |  |  |  | A-G 70.59 |  |  | Lin et al., 2021 |
| Du et al. | - | 2017-19 | PS | Zhejiang |  |  |  |  |  |  |  |  |  |  |  |  |  |  |  | A2063/2064G 85.7 |  |  | Du et al., 2020 |
| Lin et al. | 213 | 2019 | sputum | Yunnan | 5.63 |  |  | 3.76 |  |  | 4.69 |  |  | 5.63 |  |  | 2.35 |  |  | A-G 1.88 | A-G 1.41, A-C 2.35 |  | Lin et al., 2022c |
| Jiang et al. | - | 2019 | PS | Shandong |  |  |  |  |  |  |  |  |  |  |  |  |  |  |  | A-G 100 |  |  | Jiang et al., 2021 |

Note: BALF- bronchoalveolar lavage fluid. PS: pharynx swab

| Supplementary Table 3. Trends of *Bordetella pertussis* resistance to macrolide in Chinese children. |
| --- |

| Authors | Number (n) | Period | Type of specimen | District | Resistance to erythromycin | | | Resistance to azithromycin | | | Resistance to Clarithromycin | | | Mutation of gene (%) | References |
| --- | --- | --- | --- | --- | --- | --- | --- | --- | --- | --- | --- | --- | --- | --- | --- |
|  |  |  |  |  | Resistant  Rate(%) | MIC 50  (ug/ml) | MIC90  (ug/ml) | Resistant  Rate(%) | MIC 50  (ug/ml) | MIC90  (ug/ml) | Resistant  Rate(%) | MIC 50  (ug/ml) | MIC90  (ug/ml) |  |  |
| Jin et al. | 16 | 2000-2007 | NS | Beijing, Tianjin, Hebei | 0 |  |  | 0 |  |  | 0 |  |  |  | (Jin et al., 2008) |
| Zhang et al. | 2 | 2011 | NS | Shandong | 100 | ＞256 | ＞256 |  |  |  |  |  |  | A2047G 100% | (Zhang et al., 2013b) |
| Wang et al. | 4 | 2012 | NS | Shanxi | 100 | ＞256 | ＞256 | 100 | ＞256 | ＞256 | 100 | ＞256 | ＞256 | A2047G 100% | (Wang et al., 2013b) |
| Wang et al. | 16 | 2012-2013 | NS (culture positive) | Shanxi | 87.5 | ＞256 | ＞256 |  |  |  |  |  |  | A2047G 87.5% | (Wang et al., 2014) |
|  |  |  | NS (PCR positive) |  |  |  |  |  |  |  |  |  |  | A2047G 85% |  |
| Li et al. | 18 | 2012-2017 | NS | Tianjin | 94.4 |  |  | 94.4 |  |  | 94.4 |  |  | A2047G 100% | (Li et al., 2018) |
| Yang et al. | 6 | 1970 | NS | Beijing | 0 | 0.094 | 0.25 | 0 | 0.032 | 0.064 | 0 | 2 | 2 |  | (Yang et al., 2015b) |
|  | 19 | 2000-2008 |  |  | 0 | 0.064 | 0.125 | 0 | 0.032 | 0.064 | 0 | 1 | 1 |  |  |
|  | 99 | 2013-2014 |  |  | 91.9 | ＞256 | ＞256 | 91.9 | ＞256 | ＞256 | 91.9 | ＞256 | ＞256 |  |  |
| Li et al. | 32 | 2013-2018 | NS | Beijing |  | ＞256 | ＞256 |  | ＞256 | ＞256 |  | ＞256 | ＞256 | A2047G 78% | (Li et al., 2019d) |
| Li et al. | 335 | 2014-2016 | NS | Beijing, Shandong, Guangdong, Zhejiang | 87.46 | ＞256 | ＞256 |  | ＞256 | ＞256 |  | ＞256 | ＞256 | A2047G 87.16% | (Li et al., 2019b) |
| Yan et al. | 154 | 2015-2017 | NS | Guangdong | 50.65 | ＞256 | ＞256 | 50.65 | ＞256 | ＞256 | 50.65 | ＞256 | ＞256 |  | (Yan, 2019) |
| Zhang et al. | 105 | 2015-2017 | NS | Guangdong | 48.6 | 0.25 | ＞256 | 48.6 | 0.19 | ＞256 | 48.6 | 1.5 | ＞256 | A2047G 48.57% | (Zhang et al., 2020) |
| Hua et al. | 126 | 2016 | NS | Zhejiang | 75.4 | ＞256 | ＞256 | 75.4 | ＞256 | ＞256 | 75.4 | ＞256 | ＞256 |  | (Hua et al., 2019) |
| Zhe et al. | 211 | 2017 | NS | Zhejiang | 68.2 |  |  | 68.2 |  |  |  |  |  |  | (Zhe et al., 2019) |
| Lin et al. | 130 | 2018-2020 | NS | Zhejiang | 75.4 | ＞256 | ＞256 | 75.4 | ＞256 | ＞256 |  |  |  |  | (Lin et al., 2022b) |
| Juan et al. | 58 | 2018-2020 | NS | Shanxi | 79.31 | ＞256 | ＞256 | 79.31 | ＞256 | ＞256 | 75.86 | ＞256 | ＞256 | A2047G 75.86% | (Juan et al., 2022) |

Supplementary Table 4. Trends of *Group A streptococcus* resistance to macrolide in Chinese children.

| Authors | Number of strains (n) | Period (year) | Type of specimens | District | Resistance to erythromycin | | | Resistance to azithromycin | | | Resistance gene(%) | | References |
| --- | --- | --- | --- | --- | --- | --- | --- | --- | --- | --- | --- | --- | --- |
|  |  |  |  |  | Resistance rate (%) | MIC50 (ug/ml) | MIC90 (ug/ml) | Resistance rate(%) | MIC 50 (ug/ml) | MIC90 (ug/ml) | *ermB*  (%) | *mefA*  (%) |  |
| Su et al. | 620 | 1988-94 | TS | Guangdong, Hubei, Jilin, Chongqing | 35.2 |  |  |  |  |  |  |  | (Su et al., 2003) |
| Dong et al. | 376 | 1993-94 | TS | Jilin, Hubei, Guangdong, Sichuan | Jilin: 75.26  Sichuan: 22.22  Hubei: 1.1  Guangzhou: 27.19 |  |  |  |  |  |  |  | (Dong et al., 1999) |
| Dong et al. | 517 |  |  | Guangdong | Country:4.4-23.8  Countryside:21.4-67.4 |  |  |  |  |  |  |  | (Dong et al., 2001) |
| Dong et al. | 47 | 2005-07 | Cultures of abscesses | Guangdong | 47.1 |  |  |  |  |  |  |  | (Deng et al., 2008) |
| Wang et al. | 265 | 2009 | Respiratory tract secretion, blood, urinal | Shanghai, Hubei, Beijing, Zhejiang, Xinjiang, Anhui, Guangdong, Gansu, Yunnan, Chongqing | 82.1 |  |  |  |  |  |  |  | (Wang et al., 2010) |
| Zhou et al. | 127 | 2004-11 | TS | Sichuan | 98.4 |  |  |  |  |  | 96.46 |  | (Zhou et al., 2014a) |
| Liang et al. | 145 | 2007 | TS | Beijing, Shanghai | 97.9 | 512 | 512 | 97.9 | 512 | 512 |  |  | (Liang et al., 2008) |
| Liu et al. | 188 | 2007 | TS | Beijing, Shanghai, Chongqing, Guangdong | 96.8 | ＞256 | ＞256 | 98.4 | ＞256 | ＞256 |  |  | (Liu et al., 2009) |
| Chang et al. | 94 | 2007-08 | TS | Beijing, Chongqing | 96.8 | 512 | 512 | 96.8 | 512 | 512 |  |  | (Chang et al., 2010) |
| Chen et al. | 58 | 2011 | TS | Shanghai | 93.1 |  |  |  |  |  |  |  | (Chen et al., 2012) |
| Ji et al. | 52 | 2003-08 | Cultures of abscesses | Beijing | 100 | ＞256 | ＞256 | 100 | ＞256 | ＞256 | 92.30 |  | (Ji et al., 2012) |
| Yin et al. | 198 | 2012-16 | TS | Tianjin |  |  |  | 96.8 |  |  |  |  | (Yin et al., 2018) |
| Ma et al. | 222 | 2005-06 | TS, blood, pus | Beijing, Shanghai, Guangdong, Chongqing |  |  |  |  |  |  | 94.71 |  | (Ma et al., 2008) |
| Feng et al. | 371 | 2005-08 | TS, blood, pus | Beijing, Shanghai, Guangdong, Chongqing |  |  |  |  |  |  | 95.8 |  | (Feng et al., 2010a) |
| Wang et al. | 71 | 2011 |  | Beijing | 100 |  |  |  |  |  |  |  | (Wang et al., 2013a) |
| Zhu et al. | 234 | 2013-19 | TS | Beijing | 98.29 |  |  |  |  |  |  |  | (Zhu et al., 2021) |
| Sun et al. | 50 | 2016 | TS | Guangdong | 96 |  |  |  |  |  |  |  | (Sun et al., 2022) |
|  | 50 | 2017 |  |  | 92 |  |  |  |  |  |  |  |  |
|  | 50 | 2018 |  |  | 96 |  |  |  |  |  |  |  |  |
|  | 50 | 2019 |  |  | 96 |  |  |  |  |  |  |  |  |
|  | 50 | 2020 |  |  | 96 |  |  |  |  |  |  |  |  |
| Tan et al. | 66 | 2017 |  | Guangdong | 96.97 |  |  |  |  |  |  |  | (Tan et al., 2019) |
| Feng et al. | 197 |  | TS, blood | Beijing, Chongqing, Guangdong |  |  |  |  |  |  | 94.41 |  | (Feng et al., 2010b) |
| Liu et al. | 72 | 2013 | TS | Shandong |  |  |  |  |  |  | 100 |  | (Liu et al., 2015) |

Note: Throat swab--TS

Supplementary Table 5. Trends of *Group B streptococcus* resistance to macrolide in Chinese children.

| Authors | Number of strains (n) | Period (year) | Type of specimens | District | Resistance to erythromycin | | | Resistance to azithromycin | | | Resistance gene() | | References |
| --- | --- | --- | --- | --- | --- | --- | --- | --- | --- | --- | --- | --- | --- |
|  |  |  |  |  | Resistance rate(%) | MIC50 (ug/ml) | MIC90 (ug/ml) | Resistance rate(%) | MIC 50 (ug/ml) | MIC90 (ug/ml) | *ermB*  *(%)* | *mefA/E*  *(%)* |  |
| Cao et al. | 10 | Before 1989 | VS, ear swabs, outer ear skin of newborn | Beijing | 0 | -- | -- | -- | -- | -- | -- | -- | Cao et al., 1989 |
| Zhang et al. | 53 | 1990-91 | VS, ear swab, NS, umbilical cord secretion | Beijing | 66.04 |  |  |  |  |  |  |  | Zhang et al., 1995 |
| Shen et al. | 193 | 1994-99 | Vaginal swabs, cervical swab | Beijing, Guangdong | Beijing  1994-1997: 0  1998: 8  1999: 16  Guangzhou :45 |  |  |  |  |  | Only *ermB*44  Only *mefA* 29  *ErmB*+*MefA* 13.3  No *ermB*+*mefA* 13.3 | | Shen et al., 2005 |
| Yang et al. | 113 | 1996-99 |  | Beijing, Guangdong | 46 |  |  |  |  |  | *ermA* and/or *ermB* 53 | | Yang et al., 2002a |
| Guo et al. | 65 | 2006 | VS, US | Zhejiang | 86.2 |  |  |  |  |  |  |  | Guo et al., 2012 |
|  | 72 | 2007 | VS, US |  | 86.1 |  |  |  |  |  |  |  |  |
|  | 99 | 2010 | VS,US |  | 84.8 |  |  |  |  |  |  |  |  |
| Lin et al. | 9 | 2008 | Urine, pus, blood, gastric juice, PE, CSF, amniotic fluid | Fujian | 77.8 |  |  |  |  |  |  |  | Lin et al., 2015 |
|  | 22 | 2009 |  |  | 78.6 |  |  |  |  |  |  |  |  |
|  | 22 | 2010 |  |  | 42.9 |  |  |  |  |  |  |  |  |
|  | 31 | 2011 |  |  | 66.7 |  |  |  |  |  |  |  |  |
|  | 46 | 2012 |  |  | 77.5 |  |  |  |  |  |  |  |  |
|  | 49 | 2013 |  |  | 93.3 |  |  |  |  |  |  |  |  |
| Li et al. | 51 | 2008 | VS, blood, urine, umbilical cord, TS, pus, sputum, gastric juice | Zhejiang | 51 |  |  |  |  |  |  |  | Li et al., 2015 |
|  | 57 | 2009 |  |  | 49.1 |  |  |  |  |  |  |  |  |
|  | 61 | 2010 |  |  | 52.5 |  |  |  |  |  |  |  |  |
|  | 61 | 2011 |  |  | 54.1 |  |  |  |  |  |  |  |  |
|  | 69 | 2012 |  |  | 52.2 |  |  |  |  |  |  |  |  |
|  | 79 | 2013 |  |  | 57 |  |  |  |  |  |  |  |  |
| Zhang et al. | 126 | 2009 | Vaginal swabs, anal swabs | Guangdong | 69.8 |  |  |  |  |  |  |  | Zhang et al., 2015b |
|  | 147 | 2010 |  |  | 66.7 |  |  |  |  |  |  |  |  |
|  | 152 | 2011 |  |  | 61.2 |  |  |  |  |  |  |  |  |
|  | 236 | 2012 |  |  | 60.6 |  |  |  |  |  |  |  |  |
|  | 162 | 2013 |  |  | 69.8 |  |  |  |  |  |  |  |  |
|  | 102 | 2014 |  |  | 67 |  |  |  |  |  |  |  |  |
| Chen et al. | 204 | 2003-07 |  | Guangdong | 47 |  |  |  |  |  | 71.1 | A：52.2  E：68.9 | Chen et al., 2010a |
| Zhao et al. | 46 | 2003-06 | Vaginal swabs, throat swab | Beijing | 8.6 |  |  |  |  |  |  |  | Zhao et al., 2007 |
| Lin et al. | 90 | 2018-20 | Blood | Guangdong | 81.11 |  |  |  |  |  |  |  | Lin et al., 2022a |
| Yu et al. | 13 | 2013-15 | Blood | Jiangxi | 61.54 |  |  |  |  |  |  |  | Yu and Hu, 2018 |
| Qu et al. | 12 | 2014-19 | Blood | Chongqing | 91.7 |  |  |  |  |  |  |  | Qu et al., 2022 |
| Chen et al. | 16 | 2011-13 | Blood | Guangdong | 62.5 |  |  |  |  |  |  |  | Chen et al., 2014b |
| Liang and Wang | 60 | 2015-18 | Blood | Zhejiang | 43.3 |  |  |  |  |  |  |  | Liang and Wang, 2019 |
| Liu et al. | 54 | 2014-20 | Blood | Zhejiang | 60.5 |  |  |  |  |  |  |  | Liu et al., 2021a |
| Xie and Liu | 53 | 2014-17 | Blood | Henan | 58.49 |  |  |  |  |  |  |  | Xie and Liu, 2020 |
| Huang et al. | 52 | 2001-17 | Blood | Shanghai | 46 |  |  |  |  |  |  |  | Huang et al., 2018 |
| Zhan et al. | 24 | 2013-17 | Blood | Jiangsu | 75 |  |  |  |  |  |  |  | Zhan et al., 2018 |
| Wang et al. | 30 | 2016-18 | Blood | Guangdong | 53.33 |  |  |  |  |  |  |  | Wang et al., 2018 |
| Liu et al. | 16 | 2016-19 | Blood | Jiangxi | 100 |  |  |  |  |  |  |  | Liu et al., 2019 |
| Li et al. | 93 | 2013-16 | Blood，CSF, synovial fluid, bone marrow | Guangdong | 60.2 |  |  |  |  |  | Only *ermB*87.5  *ErmB*+*MefA* 3.6 | | Li et al., 2019a |
| Huang et al. | 49 | 2010-14 | blood, CSF | Guangdong | 63.27 |  |  |  |  |  |  |  | Huang et al., 2016 |
| Li et al. | 20 | 2015-19 | Blood | Henan | 85 |  |  |  |  |  |  |  | Li et al., 2020a |
| Lin et al. | 96 | 2016-18 | invasive infectious newborn | Fujian, Shanghai | 79.3-91.0 |  |  |  |  |  |  |  | Lin et al., 2019 |
| Li et al. | 46 | 2013-16 | blood, CSF | Guangdong | 76.1 |  |  |  |  |  |  |  | Li et al., 2018c |
| Zhong et al. | 108 | 2011-14 | blood, CSF, gastric juice, urine | Guangdong | 78.9 |  |  |  |  |  |  |  | Zhong et al., 2015 |
| Lei et al. | 20 | 2006-14 | blood, CSF | Tianjin | 75 |  |  |  |  |  |  |  | Lei et al., 2015 |
| Wang et al. | 5 |  | blood, CSF | Beijing | 80 |  |  |  |  |  |  |  | Wang et al., 2019b |
| Zhang et al. | 35 | 2009-12 | blood, CSF | Guangdong | 41 |  |  |  |  |  |  |  | Zhang et al., 2015a |
| Wang et al. | 40 | 2008-13 | invasive GBS isolates | Beijing, Guangdong | 92.5 | ≥256 | ≥256 | 97.5 | ≥256 | ≥256 | Only *ermB* 73  Only *mefA* 5.4  *ErmB*+*MefA* 21.6 | | Wang et al., 2015a |
| Wang et al. | 56 | 2012-13 | Vaginal swabs, anal swabs | Beijing | 78.6 | ≥16 | ≥256 | 87.5 | ≥256 | ≥256 | Only *ermB* 43.2  Only *mefA* 22.7  *ErmB*+*MefA* 29.5  No *ermB*+*mefA* 4.5 | | Wang et al., 2015b |
| Wang et al. | 1552 | 2013-19 | Vaginal swabs, anal swabs | Zhejiang |  |  |  | 99.7 |  |  |  |  | Wang et al., 2020 |
| Du | 62 | 2017-21 | Invasive GBS isolates | Jiangxi | 83.9 |  |  |  |  |  | 86.5 | 44.2 | Du, 2022 |

CSF-cerebrospinal fluid, TS-throat swabs, NS- nose swabs， VS: Vaginal secretion，US：urethral secretions, PE: pleural effusion

Supplementary Table 6. Trends of *Staphylococcus aureus* resistance to macrolide in Chinese children.

| SA | Number of strains | period | Type of specimens | District | erythromycin | | | azithromycin | | | References |
| --- | --- | --- | --- | --- | --- | --- | --- | --- | --- | --- | --- |
|  |  |  |  |  | Resistance rate(%) | MIC 50  (ug/ml) | MIC90  (ug/ml) | Resistance rate(%) | MIC 50  (ug/ml) | MIC90  (ug/ml) |  |
| Huang and Liu | 109 | 1979-88 | blood, pus, urine | Chongqing | 65.5 |  |  |  |  |  | Huang and Liu, 1990 |
| Zhang et al. | 1297 | 1988-95 | blood, bone marrow fluid | Anhui | 65.8 |  |  |  |  |  | Zhang et al., 1998 |
| Jin et al. | 58 | 1994-96 | lower respiratory tract secretion | Shanghai | 75.6 |  |  |  |  |  | Jin et al., 1999 |
| Li and Xiong | 25  19  17 | 1995  1997  1999 | sputum and lower respiratory tract secretion | Guangdong | 72  94.7  100 |  |  |  |  |  | Li and Xiong, 2001 |
| Zhou | MRSA:58 | 1990-95 | Blood, pus, CSF | Guangdong | MRSA:100 |  |  |  |  |  | Zhou, 1997 |
| Fan et al. | 41  64  30  135  51 | 1993  1994  1995  1996  1998 | invasive and non-invasive | Beijing | 80.5  79.7  83.3  85.9  90.3  MRSA:97.1  MSSA:81.0 |  |  |  |  |  | Fan et al., 2000 |
| Ma et al. | 212 | 1998 | Skin secretions | Beijing | 87.7 |  |  |  |  |  | Ma et al., 2000 |
| Guo et al. | 76 | 1995-97 | blood | Shanxi | 42.1 |  |  |  |  |  | Guo et al., 2000 |
| Duan et al. | 62 | Before 2000 | blood | Jiangxi | 63 |  |  |  |  |  | Duan et al., 2000 |
| Huang and Chen | 200 | 1998-99 | blood | Zhejiang | 76 |  |  |  |  |  | Huang and Chen, 2001 |
| Xu and Shao | 1293 | 1991-00 | blood | Zhejiang | 70.4 |  |  |  |  |  | Xu and Shao, 2002 |
| Wang et al. | 7835 | 2000-06 | invasive and non-invasive | Beijing,Shanghai,Guangzhou,Chongqing | 66.39 |  |  |  |  |  | Wang et al., 2008 |
| Xia et al. | 780 | 2006-10 | sputum | Jiangsu | 84.5 |  |  |  |  |  | Xia et al., 2012 |
| Zhao et al. | 2245 | 2005-10 | sputum and blood | Throughout the country | 71 |  | >256 |  |  |  | Zhao et al., 2012a |
| Chen et al. | 2525 | 2008-14 | sputum and blood, skin soft tissue | Jiangxi | 64.9 |  |  |  |  |  | Chen et al., 2017 |
| Hua et al. | 145 | 2001-02 | Sputum, throat swab, blood, vaginal secretion, conjunctival sac secretion, CSF and pus | Hangzhou | 37.93 |  |  |  |  |  | Hua et al., 2003 |
| Long and Wang | 106 | 2009-10 | Blood, sputum, pus, feces, secretions | Qinghai | 24.5 |  |  |  |  |  | Long and Wang, 2012 |
| Sun | 61 | 2007  2008  2009 | blood | Zhejiang | 11.1  20.0  25.9 |  |  |  |  |  | Sun, 2011 |
| Shi and Jian | MRSA：26  MSSA：32 | 2006-08 | blood | Hubei | MRSA: 88.5  MSSA:46 |  |  |  |  |  | Shi and Jian, 2010 |
| Wang | 152 | 2009-11 | Invasive and non-invasive | Xinjiang | MRSA:83.7  MSSA:30.3 |  |  |  |  |  | Wang, 2013 |
| Zhang and Jin | MRSA:34  MSSA:478 | 2010-11 | Blood, sputum, pus, secretions | Hubei |  |  |  | MRSA:19.0  MSSA:58.9 |  |  | Zhang and Jin, 2013 |
| Zhang et al. | MRSA:27  MSSA:53 | 2008-10 | Umbilical cord secretion | Hunan | MRSA:77.78  MSSA:54.72 |  |  |  |  |  | Zhang et al., 2013a |
| Wang et al. | MRSA:43  MSSA:202  Newborns:27  Infants:218 | 2007-09 | Invasive and non-invasive | Zhuhai | MRSA:93  MSSA:62.2  Newborns:59.2  Infants:68.6 |  |  |  |  |  | Wang et al., 2011b |
| Hu and Xia | MRSA/MSSA：35/576  75/627  135/196  204/231 | 2004  2005  2006  2007 | Sputum, throat swabs, urine, blood, secretions, puncture fluid | Wuhan | MRSA/MSSA：  94.3/45  97.3/51  80.0/61.2  80.9/63.2 |  |  |  |  |  | Hu and Xia, 2009 |
| Wang et al. | MRSA: 47 | 2008-09 | lower respiratory tract secretion | Liaoning,Zhejiang,Guangdong,Chongqing,Shanghai | MRSA: 97.9 | >256 | >256 |  |  |  | Wang et al., 2011a |
| Huang et al. | 425 | 2010-12 | sputum, blood, urine, feces, pharyngeal swab | Heilongjiang | 67.29 |  |  |  |  |  | Huang et al., 2014 |
| Zhai et al. | 60 | 2012-14 | blood | Liaoning | 81.7 |  |  |  |  |  | Zhai et al., 2016 |
| Bao et al. | 124 | 2018-20 | blood | Beijing | 73.39 |  |  |  |  |  | Bao et al., 2021 |
| Wu et al. | 54  56  57  62  68 | 2013  2014  2015  2016  2017 | pus, secretions | Hainan | 64.8  66.0  68.4  72.6  77.9 |  |  |  |  |  | Wu et al., 2019 |
| Li et al. | 448  MRSA:42  MSSA:406 | 2011-14 | Sputum, throat swabs | Nanjing | 58.0  MRSA:76.2  MSSA:56.2 |  |  |  |  |  | Li et al., 2016 |
| Ding and Li | 110  65  82 | 2018  2019  2020 | Sputum, secretions, pus, blood | Neimenggu | 79.41  81.69  84.21 |  |  |  |  |  | Ding and Li, 2022 |
| Chi et al. | MRSA:78  MSSA:358 | 2014-17 | Invasive and non-invasive | Chongqing | MRSA:80.77  MSSA:53.45 |  |  |  |  |  | Chi et al., 2018 |
| Fu et al. | MRSA：11128  MSSA：20667  total：31795 | 2016-20 | Invasive and non-invasive | Throughout the country | MRSA: 78.2  MSSA: 51.9  total: 61.1 |  |  |  |  |  | Fu et al., 2021 |
| He et al. | MSSA：54  MRSA：35 | 2011-16 | sputum | Guangdong | MSSA: 29.6  MRSA :81.8 |  |  | MSSA 22.2  MRSA 83.3 |  |  | He et al., 2017 |
| Huang et al. | MRSA:194  MSSA:453 | 2011-12 | Invasive and non-invasive | Guangdong | MRSA:81.35  MSSA:61.52 |  |  |  |  |  | Huang et al., 2015a |
| Xiao et al. | Total:57  MRSA:14  MSSA:43 | 2018-19 | Blood, CSF, deep sputum, BALF, pus | Guangxi | Total:29.8  MRSA:71.4  MSSA:16.3 |  |  |  |  |  | Xiao et al., 2021 |
| Hu et al. | CA-MRSA70  HA-MRSA45  HACO-MRSA 68 | 2014 | blood | Shenzhen | CA-MRSA:81.4  HA-MRSA:86.7  HACO-MRSA:86.8 |  |  |  |  |  | Hu et al., 2016 |
| Chen et al. | CAP-MSSA：329  CA-MRSA：105  HA-MSSA：808  HA-MRSA：658 | 2015-17 | Invasive and non-invasive | Throughout the country | CA--MSSA:60  CA--MRSA:86  HA--MSSA :55  HA--MRSA: 82 |  |  |  |  |  | Chen et al., 2022 |
| Zhou et al. | Total:86  MRSA:20  MSSA:66 | 2018-20 | Ear secretion | Guangxi | Total:67.4  MRSA:100  MSSA:57.6 |  |  |  |  |  | Zhou et al., 2022 |
| Min et al. | MRSA:296  MSSA:408 | 2014-17 | Sputum, secretions, pus, blood | Jiangxi | MRSA:87.5  MSSA:56.8 |  |  |  |  |  | Min et al., 2019 |
| Zhao et al. | MRSA:149  MSSA:115 | 2014-16 | Sputum, secretions, pus, blood, BALF | Anhui | MRSA:91.3  MSSA:66.1 |  |  |  |  |  | Zhao et al., 2020 |
| Deng et al. | 244 | 2009-11 | Invasive and non-invasive | Sichuan | 57.79 |  |  |  |  |  | Deng et al., 2013 |
| Deng et al. | 257 | Before 2011 | Nasal vestibular swab | Chengdu | 74.71 |  |  |  |  |  | Deng et al., 2012 |
| Ning et al. | 121 | 2011-12 | Pus | Zhejiang | 98.35 | >256 | >256 |  |  |  | Ning et al., 2014 |
| Ran et al. | 300 | 2015-16 | Pus | Chengdu | 71 | 256 | >256 |  |  |  | Ran et al., 2017 |
| Zhang et al. | Inhaled:24  Blood-borne:20 | 2008-13 | Pleural effusion, blood | Chongqing | Inhaled:74  Blood-borne:80 |  |  |  |  |  | Zhang et al., 2014a |
| Li et al. | Class 1 integrons (+) ：236  Class 1 integrons (-)：364  adult：153  child：447 | 2018-19 | sputum、blood、urine、feces、pus | Guangdong | Class 1 integrons (+) : 50.85  Class 1 integrons (-) ：39.29  adult：36.6  child：46.31 |  |  |  |  |  | Li et al., 2020b |
| Ma et al. | Child:102  Adult:39 | 2004 | Invasive and non-invasive | Hangzhou | Child:49.0  Adult:89.8 |  |  |  |  |  | Ma et al., 2007 |
| Chen et al. | *Mec*A+：66  *Mec*A-：120 | 2012 | sputum | Guangdong | *Mec*A+: 80.3  *Mec*A-: 35 |  |  |  |  |  | Chen et al., 2014a |
